# Chinese herbal medicine for psoriasis: Protocol for an overview of systematic reviews

**DOI:** 10.1097/MD.0000000000022400

**Published:** 2020-09-25

**Authors:** Jie Zhang, Qianying Yu, Li Peng, Wenxia Lin, Yuesi Qin, Ying He, Jing Guo, Min Xiao, Mingling Chen

**Affiliations:** a Affiliated Hospital of Chengdu University of Traditional Chinese Medicine; b Chengdu Integrated TCM & Western Medicine Hospital, Chengdu, Sichuan Province, P. R. China.

**Keywords:** AMSTAR 2, Chinese herbal medicine, GRADE, overview, PRISMA, psoriasis, ROBIS

## Abstract

**Background:**

Psoriasis is a chronic recurrent dermatological disease that patents always suffer from different comorbidities. Chinese herbal medicine (CHM) has been commonly used in the treatment of psoriasis for a long history. Previous systematic reviews (SRs)/meta-analyses (MAs) have shown that CHM may benefit patients with psoriasis. This overview aims to summarize the evidence from published SRs/MAs for clinical application and to provide several directions for future researches.

**Methods:**

Nine electronic databases (Medline, Embase, Cochrane Library, AMED, CINAHL, CBM, CNKI, VIP Database, Wanfang Databases) will be searched from their inceptions to September 2020 without language restrictions. At least 2 reviewers will independently conduct the study selection, data extraction, and quality assessment. The methodological quality, risk of bias, reporting quality, and evidence quality will be respectively evaluated by the Assessing the Methodological Quality of Systematic Reviews 2 (AMSTAR 2), the Risk of Bias in Systematic Reviews, the Preferred Reporting Items for Systematic Reviews and Meta-Analyses (PRISMA), and the Grading of Recommendations, Assessment, Development and Evaluation (GRADE) system.

**Results:**

The results of this overview will be submitted to a peer-reviewed journal for publication.

**Conclusions:**

We expect to compile current evidence from published SRs/MAs of CHM for patients with psoriasis in an accessible and useful document.

**Ethics and dissemination:**

This study is a protocol for an overview of SRs/MAs that did not involve individual data. Thus, ethical approval is not required.

**OSF Registration number:**

DOI 10.17605/OSF.IO/VC654

## 1 Introduction

Psoriasis, a common chronic skin disease, is characterized by erythematous, pruritic plaques covered with silvery scales and is considered a multisystemic disease that patients often suffer from many different disorders, including hypertension, type 2 diabetes, coronary heart disease, and so on.^[1]^ In addition to the physical burden, patients always have a substantial economic burden and psychological burden. Thus, they often report the poor quality of life.^[2–4]^ In adults, the incidence of psoriasis ranged from 30.3 per 100,000 person-years in Taiwan to 321.0 per 100,000 person-years in Italy, and the prevalence varied from 0.14% in east Asia to 1.99% in Australasia, indicating that the incidence and prevalence of psoriasis have increased in recent years.^[5]^ Due to the heavy disease burden and high prevalence, psoriasis has become a major health problem, which means that more attention should be paid to the management of psoriasis.

With a high rate of recurrence, the pathological mechanism of psoriasis is not yet clear but is thought to result from a confounding combination of genes and the environment. The 5 therapies were recommended by the international guidelines that consist of topical therapy, phototherapy, conventional systemic therapy, small molecule inhibitor, and biologic.^[6,7]^ Because of the various side-effects and high costs, widespread treatment dissatisfaction was found in patients who used conventional systemic therapy or biologic for a long time.^[4]^ Thus, patients often select complementary therapy as an alternative option or as an add-on treatment.

Chinese herbal medicine (CHM) is an important component of complementary therapies that have been applied to treat psoriasis for many years in China. Plenty of previous systematic reviews and meta-analyses had been conducted to evaluate the efficacy and safety of CHM, and they showed that both oral and external CHM may benefit the patients with psoriasis.^[8–10]^ However, some systematic reviews (SRs)/meta-analyses (MAs) reported that the evidence supporting the effectiveness of CHM is insufficient.^[11,12]^ Besides, no critically designed overview has been performed to assess the reporting and methodological quality of the published SRs/MAs so far. Therefore, this overview aims to evaluate the SRs/MAs of CHM for psoriasis and to summarize the evidence for clinical application. Moreover, we expect that our overview will provide several directions for future research.

## 2 Methods

### 2.1 Protocol and registration

The protocol of this overview has been registered on OSF (registration number: DOI 10.17605/OSF.IO/VC654). This overview of systematic reviews will be reported according to the Preferred Reporting Items for Overviews of Systematic Reviews including the harms checklist (PRIO-harms).^[13]^

### 2.2 Inclusion criteria

#### 2.2.1 Types of studies

The SRs/MAs of randomized controlled trials (RCTs) that evaluate the efficacy of CHM for the treatment of psoriasis will be included. Reviews with the most recent and comprehensive studies will be included if the same or similar original studies exist.

#### 2.2.2 Types of participants

Participants should be diagnosed as psoriasis by any international or domestic diagnostic criteria. There will be no restrictions on gender, age, ethnicity, duration, and stage of the disease.

#### 2.2.3 Types of interventions

Reviews will include an experimental group that is treated with any internal or external therapies of CHM or CHM combined with other therapies, regardless of the dosage form (e.g., Chinese patent medicine, Chinese medicine decoction, or injection). The control group treated with comfort therapy (e.g., placebo or blank control) or other therapies (e.g., Western medicine, acupuncture, or other nonpharmacological therapies) will be included.

#### 2.2.4 Types of outcome measures

The primary outcome is the total effective rate measured by the psoriasis area and severity index (PASI) score decline rate. Secondary outcomes include the mean change of PASI, quality of life assessed by the dermatology life quality index or 36-item short form health survey (SF-36), the itching index assessed by itching evaluation scale, traditional Chinese medicine (TCM) syndrome score, adverse events, and the recurrent rate.

### 2.3 Exclusion criteria

Duplicated publications, non-RCT SRs/MAs, network meta-analysis, conference abstracts, comments, overviews, protocols, SRs/MAs whose data cannot be extracted, and SRs/MAs whose objects are diagnosed as psoriasis with arthritis or other comorbidities (such as metabolic syndrome, cardiovascular disorders, depression, cancer, etc.) will be excluded. Studies on the nonmajor intervention of CHM (such as acupuncture, massage, qigong, Tai qi, etc.) in the experiment group or studies on CHM as an intervention in the control group will be also excluded.

### 2.4 Search strategy

Five international electronic databases (Medline, Embase, Cochrane Library, AMED, CINAHL) and 4 Chinese electronic databases (Chinese Biomedical Databases (CBM), China National Knowledge Infrastructure (CNKI), VIP Journals Database, Wanfang Databases) will be searched from their inceptions to September 2020 without language restrictions. The search terms will be used as follows: psoriasis, psoriases, traditional Chinese medicine, Chinese medicine, patent medicine, proprietary medicine, Chinese herbal drugs, Chinese material medica, Chinese herbal bath, systematic review, meta-analysis. The search strategy in Medline is listed in Table 1 and is modified to suit different databases. Besides, the reference lists of the included reviews, protocol registries, conference abstracts, and guidelines about psoriasis treated with CHM will be also screened to identify missing SRs/MAs.

**Table 1:**
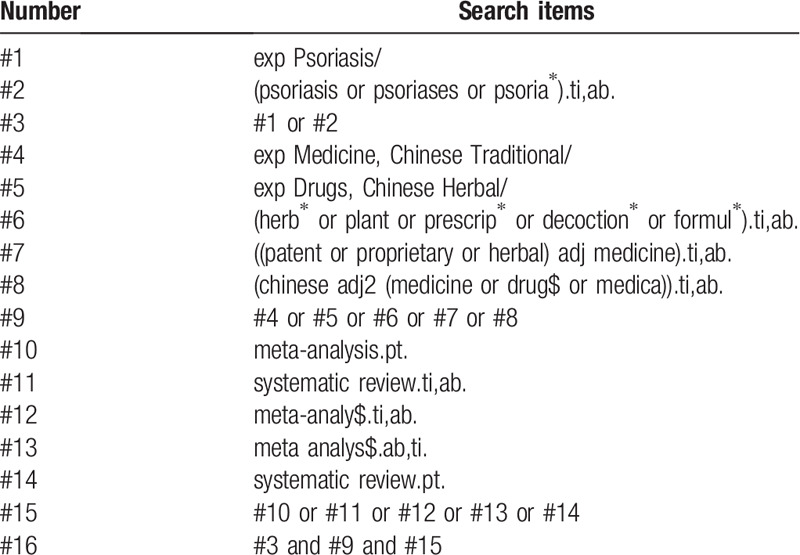
The search strategy used in Medline (via Ovid).

### 2.5 Selection of SRs/MAs

**Figure 1:**
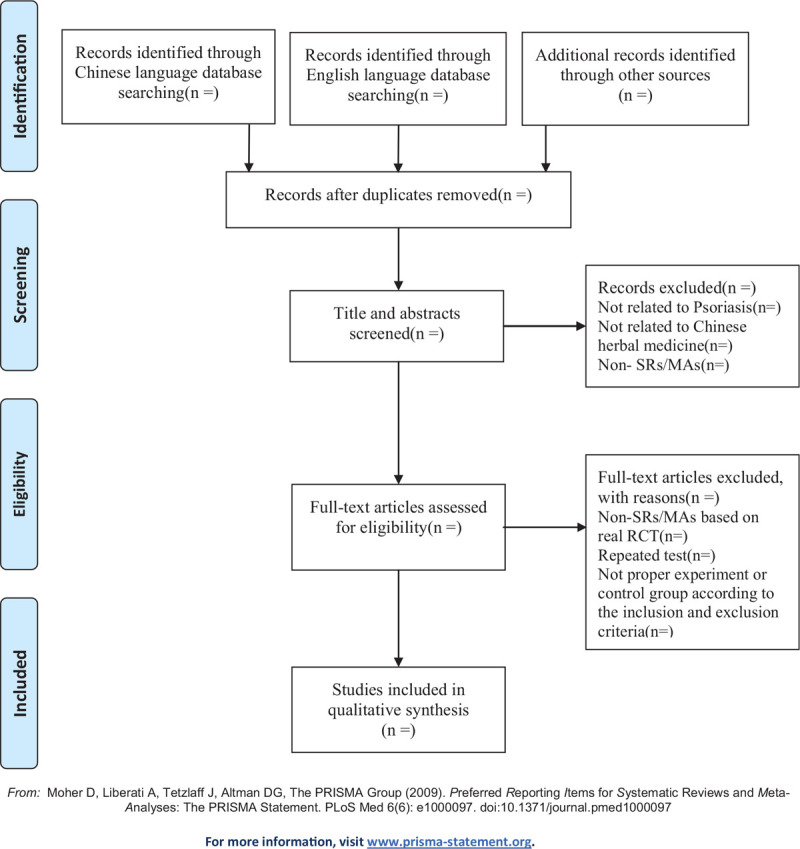
Flow diagram of the study selection process.

All the retrieved articles will be imported into the EndNote X9 software and the duplicate publications will be excluded. Two authors will independently scan the titles and abstracts of the searched articles for potentially relevant SRs/MAs. Full-text articles will be downloaded for further assessment according to the inclusion and exclusion criteria. Any disagreement will be resolved by discussion or consultation with the third author if a consensus is not reached. The flow diagram of the study selection process is presented in Figure 1.

### 2.6 Data extraction

Two authors will independently perform the data extraction following a predefined data collection form by using Excel 2017 and cross-checked each other's extracted data to avoid mis-entry. Discrepancies will be judged by the third author. The primary trials will be accessed if the information in SRs/MAs is unclear. The following data from the included SRs/MAs will be extracted: the first author, country, title, publication year, the number of included studies, sample size, diagnostic criteria, details of interventions in treatment and control groups, outcome measures, quality assessment methods, and main conclusions. The corresponding authors of the included SRs/MAs or original studies will be contacted to obtain missing data.

### 2.7 Quality assessment of SRs/MAs

Two qualified authors who are trained in the Chinese Cochrane Center will independently conduct the quality assessment of included SRs/MAs and cross-check the results after evaluation. Any disagreement will be resolved by team discussion.

#### 2.7.1 Assessment of methodological quality

Assessing the Methodological Quality of Systematic Reviews 2 (AMSTAR 2),^[14]^ a popular instrument for appraising SRs will be applied to assess the methodological quality of included SRs/MAs. AMSTAR 2 contains 16 items, and 7 of them are critical domains (items 2, 4, 7, 9, 11, 13, 15), which can critically affect the validity of the review and its conclusions. Each item is judged as “yes,” “partial yes,” and “no.” The tool divides overall confidence of the review results into 4 levels: high, moderate, low, and critically low.

#### 2.7.2 Assessment of risk of bias

The Risk of Bias in Systematic Reviews (ROBIS)^[15]^ will be applied to assess the risk of bias of included SRs/MAs. ROBIS is completed in 3 phases: Phase 1 evaluates the relevance between target question and proposed question; Phase 2 identifies concerns with the SR process through 4 domains: “study eligibility criteria,” “identification and selection of studies,” “data collection and study appraisal,” and “synthesis and findings”; Phase 3 judges the overall risk of bias in the interpretation of results in SRs and whether this considered limitations identified in any of the phase 2 domains. Answers to the signal questions are categorized as “yes,” “probably yes,” “no,” “probably no,” or “no information.” The risk of bias is judged as “high,” “low,” or “unclear.”

#### 2.7.3 Assessment of reporting quality

The Preferred Reporting Items for Systematic Reviews and Meta-Analyses (PRISMA),^[16]^ which can be useful for critical appraisal of published SRs/MAs, will be applied to assess the reporting quality of included SRs/MAs. The PRISMA statement consists of a 27-item checklist and a 4-phase flow diagram, including 7 aspects of SRs/MAs (title, abstract, introduction, methods, results, discussion, and funding). Each item is described with “yes” when the content of the item is reported adequately, “partially reported” when the content is reported inadequately, or “no” when none content is reported.

#### 2.7.4 Assessment of quality of evidence

The Grading of Recommendations, Assessment, Development and Evaluation (GRADE) system^[17]^ will be applied to assess the quality evidence of included SRs/MAs. Although evidence based on RCTs begins as high-quality evidence, the quality of evidence can also be decreased by 5 elements that include limitations in study quality, the inconsistency of results, uncertainty about the directness of evidence, imprecise or sparse data, and high risk of reporting bias. The grade of evidence is categorized into 4 levels: high, moderate, low, and very low.

### 2.8 Data synthesis

A narrative description of characteristics in the included SRs/MAs will be shown in the table. The results of SRs/MAs will be summarized as the risk ratio or odds ratio for the dichotomous data, and as the standard mean difference or weighted mean difference for the continuous data with 95% confidence intervals. If the data is unsuitable for pooling due to the substantial heterogeneity, the narrative synthesis will be conducted. The results of AMSTAR 2, ROBIS, PRISMA, and the GRADE will be presented in tabulation and figures.

## 3 Discussion

CHM has been widely used in the treatment of psoriasis for a long history. Growing evidence indicates that CHM may have considerable therapeutic effects on psoriasis. However, the reporting and methodological quality of SRs/MAs have not been evaluated, which is an indispensable step before the treatment recommendations were presented.^[18,19]^ To the best of our knowledge, this study will be the first overview to assess the published SRs/MAs of CHM for psoriasis. What we expect is that the results of this overview will give patients and doctors more information about the credibility of current evidence to make decisions, and will provide some directions for future research.

## Author contributions

**Conceptualization:** Jie Zhang.

**Data curation:** Jie Zhang, Qianying Yu, Li Peng, Wenxia Lin, Ying He.

**Formal analysis:** Jie Zhang, Qianying Yu.

**Funding acquisition:** Jing Guo, Mingling Chen.

**Investigation:** Yuesi Qin, Jing Guo, Min Xiao.

**Methodology:** Jie Zhang, Qianying Yu, Li Peng.

**Supervision:** Mingling Chen, Min Xiao, Jing Guo.

**Writing – original draft:** Jie Zhang.

**Writing – review & editing:** Mingling Chen, Jing Guo, Min Xiao.
